# Integrating Option Grid Patient Decision Aids in the Epic Electronic Health Record: Case Study at 5 Health Systems

**DOI:** 10.2196/22766

**Published:** 2021-05-03

**Authors:** Peter Scalia, Farhan Ahmad, Danielle Schubbe, Rachel Forcino, Marie-Anne Durand, Paul James Barr, Glyn Elwyn

**Affiliations:** 1 Dartmouth College Lebanon, NH United States; 2 EBSCO Health Boston, MA United States

**Keywords:** shared decision making, patient decision aids, electronic health record, implementation, HL7 SMART on FHIR

## Abstract

**Background:**

Some researchers argue that the successful implementation of patient decision aids (PDAs) into clinical workflows depends on their integration into electronic health records (EHRs). Anecdotally, we know that EHR integration is a complex and time-consuming task; yet, the process has not been examined in detail. As part of an implementation project, we examined the work involved in integrating an encounter PDA for symptomatic uterine fibroids into Epic EHR systems.

**Objective:**

This study aims to identify the steps and time required to integrate a PDA into the Epic EHR system and examine facilitators and barriers to the integration effort.

**Methods:**

We conducted a case study at 5 academic medical centers in the United States. A clinical champion at each institution liaised with their Epic EHR team to initiate the integration of the uterine fibroid Option Grid PDAs into clinician-facing menus. We scheduled regular meetings with the Epic software analysts and an expert Epic technologist to discuss how best to integrate the tools into Epic for use by clinicians with patients. The meetings were then recorded and transcribed. Two researchers independently coded the transcripts and field notes before categorizing the codes and conducting a thematic analysis to identify the facilitators and barriers to EHR integration. The steps were reviewed and edited by an Epic technologist to ensure their accuracy.

**Results:**

Integrating the uterine fibroid Option Grid PDA into clinician-facing menus required an 18-month timeline and a 6-step process, as follows: task priority negotiation with Epic software teams, security risk assessment, technical review, Epic configuration; troubleshooting, and launch. The key facilitators of the process were the clinical champions who advocated for integration at the institutional level and the presence of an experienced technologist who guided Epic software analysts during the build. Another facilitator was the use of an emerging industry standard app platform (Health Level 7 Substitutable Medical Applications and Reusable Technologies on Fast Healthcare Interoperability Resources) as a means of integrating the Option Grid into existing systems. This standard platform enabled clinicians to access the tools by using single sign-on credentials and prevented protected health information from leaving the EHR. Key barriers were the lack of control over the Option Grid product developed by EBSCO (Elton B Stephens Company) Health; the periodic Epic upgrades that can result in a pause on new software configurations; and the unforeseen software problems with Option Grid (ie, inability to print the PDA), which delayed the launch of the PDA.

**Conclusions:**

The integration of PDAs into the Epic EHR system requires a 6-step process and an 18-month timeline. The process required support and prioritization from a clinical champion, guidance from an experienced technologist, and a willing EHR software developer team.

## Introduction

### Background

Researchers have argued that the successful implementation of patient decision aids (PDAs) into clinical workflows depends on their integration into electronic health records (EHRs) [1,2]. The task of integrating third-party tools into EHRs is complex [3]. Security concerns dominate the challenge, as institutions have become reliant on EHRs to manage key operational workflows [3]. Third-party software that brings external connections and URL links to EHRs is subject to extensive scrutiny [4]. Updates to either the EHR or linked third-party products are perennial concerns, given the cost of downtime or system failure.

Many software vendors provide system-wide EHR software (eg, Epic, Cerner, or Allscripts) [5]. However, there are major differences between the same EHR product when installed at different health care institutions: this is because they are tailored to organizational and clinical preferences and integrated with other ancillary software [6]. Clinicians also differ in how and when EHRs are used with patients [7]. These uses can include showing images or test results and sending health information via the patient portal [8]. Although some common integration processes can be identified, solutions cannot be replicated from one institutional setting to others and tailoring is always required.

PDAs provide evidence-based information in a comparative format to facilitate shared decision-making, in which patients and clinicians are supported when making informed decisions together [9]. PDAs serve as catalysts to engage patients in decision-making processes and can be used before, during, and after clinical encounters [10,11]. Recent systematic reviews and meta-analyses have shown that PDAs increase knowledge about options and reduce decisional conflict, thereby helping patients make decisions that align with their preferences [12,13]. Despite improving a range of outcomes, their implementation in the clinical workflow remains a challenge [14,15]. Given the widespread adoption of EHRs and clinicians’ reliance on them [16], many have presumed that integrating PDAs into EHRs will lead to their increased use in clinical practice [2,17]. However, this presumption has not yet been tested at scale.

Studies that have evaluated the integration of PDAs in EHRs have focused on measuring their use by clinicians [1,18-22], measuring their impact on patient outcomes [23-25], or user testing the tool to improve the navigation and design in the EHR system [26-29]. The integration of 2 PDAs, namely the Statin Choice and Diabetes Medication Choice tools, in the EHR at the Mayo Clinic led to their increased use [18,20,21]. Coylewright et al [1] also demonstrated an increased use and observed that adoption rates of an EHR-based *HealthDecision* tool steadily increased over an 8-year period, with a *high rate of sustained implementation after the fifth use*.

Nevertheless, we were only able to identify a few examples of PDAs being integrated into EHR systems [1,18-29]. Anecdotally, researchers and practitioners recognize that embedding PDAs in EHRs is a complex and time-consuming process; however, we could not identify the literature that described the required processes. Therefore, we lack an understanding of *how* best to integrate these tools into EHRs, and the steps required, especially given the recent development of new interoperability standards [30]. An opportunity arose to address this research gap as part of a project to implement the uterine fibroids Option Grid PDA at 5 health care institutions in the United States (Uterine Fibroids Options [UPFRONT] study) [31].

### Objectives

The aims of this work are to (1) identify the steps and the time required to integrate an Option Grid PDA into the Epic EHR system and (2) examine facilitators and barriers to the integration effort. We hypothesize that some institutions will successfully integrate the Option Grid PDA into Epic as part of a multistep, time-intensive process.

## Methods

### Design

As part of a Patient-Centered Outcomes Research Institute (PCORI)–funded, stepped-wedge implementation trial, we asked each participating institution to integrate Option Grid PDAs into their Epic EHR systems. Despite the stepped-wedge design, we began the integration effort at all institutions almost immediately upon receiving funding to provide ample opportunity to complete the process ahead of the active implementation phase of the broader trial, which is when clinicians would be expected to use Option Grid with their patients. Successful integration was defined as the completion of changes to the Epic system that allowed clinicians to easily access an external website that provides access to both interactive and PDF versions of the uterine fibroid Option Grid PDAs [31]. Facilitators are key elements or factors that enable a successful integration. To examine the processes required, we adopted an exploratory case study design and collected data by recording relevant meetings and taking field notes [32]. We analyzed our conversations with the clinical champions, their research teams, and Epic software analysts from various departments (such as compliance, risk management, and information security) at each institution. Our case study was reported using the checklist by Rodgers et al (Multimedia Appendix 1) [33]. The Dartmouth College Committee for the Protection of Human Subjects (approval number: STUDY00031464) granted ethical approval for our study.

### Settings

The implementation study took place in the following institutions: (1) Dartmouth-Hitchcock Medical Center in Lebanon, New Hampshire; (2) Barnes-Jewish Hospital in St. Louis, Missouri; (3) Montefiore Medical Center in Bronx, New York; (4) Brigham and Women’s Hospital in Boston, Massachusetts; and (5) Mayo Clinic in Rochester, Minnesota. Each of these institutions had installed the Epic EHR product at different times. Our case study is based on our efforts to integrate the Option Grid into the Epic EHR system at these 5 institutions. Table 1 provides a brief description of each institution’s Epic experience, expertise, and infrastructure.

**Table 1:** Description of each institution’s Epic experience, expertise, and infrastructure.

Institution	Date of Epic adoption	Number of Epic software analysts^a^	Any previous experience with third-party software integration?	Does the clinical champion have experience with using patient decision aids?
Dartmouth-Hitchcock Medical Center	April 2011	170	Yes	Yes
Barnes-Jewish Hospital	June 2017	150	Yes	No
Montefiore Medical Center	April 2015 to June 2016	200	Yes	No
Brigham and Women’s Hospital	June 2015	Unknown	Yes	Yes
Mayo Clinic	May 2018	300	Yes	No

^a^Estimated number.

### Option Grid PDA

The Option Grid PDA for symptomatic uterine fibroids is part of a suite of tools developed and updated by EBSCO (Elton B Stephens Company) Health, a commercial entity that provides clinical decision support for health care organizations [34]. Our collaboration with EBSCO Health for developing and maintaining the uterine fibroids PDA came at no cost to the research effort. The uterine fibroid tool compares 7 treatment options: (1) watch and wait, (2) medicine with hormones, (3) medicine without hormones, (4) embolization, (5) endometrial ablation, (6) myomectomy, and (7) hysterectomy. The tool is available in English and Spanish for the following 2 formats: text-only and text accompanied by pictures (Picture Option Grid). For the study duration, each participating institution was granted access to the entire suite of 30 Option Grid tools.

**Figure 1:**
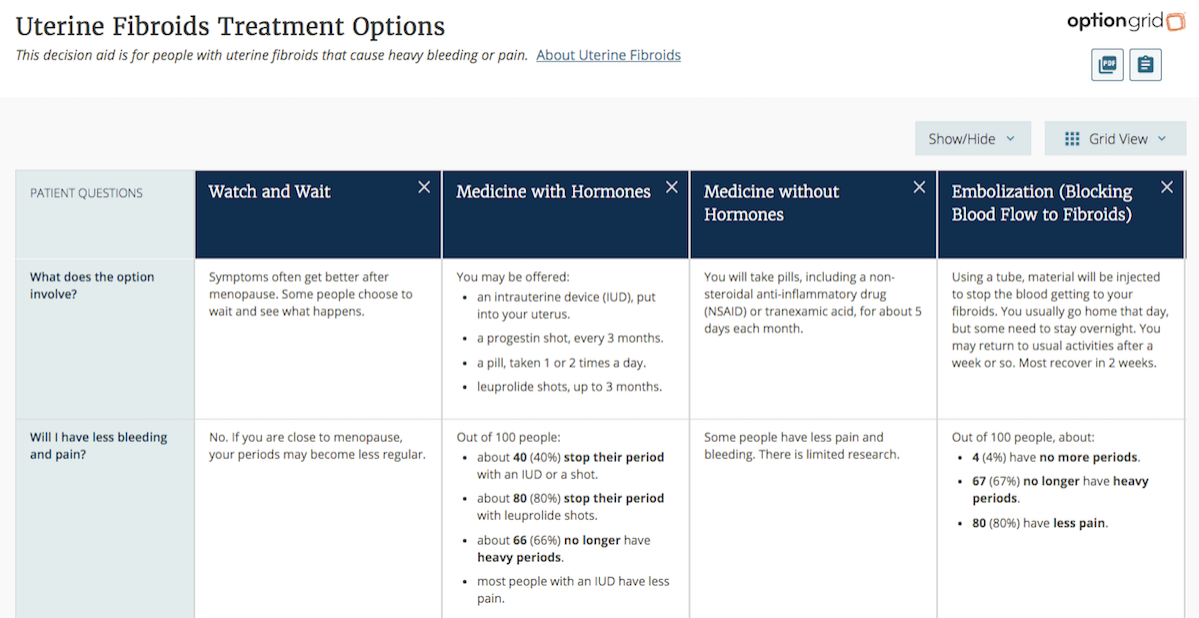
Snapshot of the text version of the uterine fibroid Option Grid patient decision aid.

When clinicians click the Option Grid button, they are first presented with the entire suite of PDAs. Once clinicians select the uterine fibroids Option Grid, they have the opportunity to select as many as 7 treatment options that are relevant to a particular patient. Once the PDA is generated, the clinician can use 3 features to document the options discussed in the encounter: print a PDF version, copy and paste a script that includes the options selected, or send a *permalink* to the patient so that they can view the Option Grid at their own convenience. The script is an optional feature that enables clinicians to document the use of the PDA and the options discussed in the EHR. The clinician must either use this feature or write a note in the EHR regarding the conversation that occurred with the PDA, as Option Grid does not exchange any information with the EHR. Figure 1 shows an example of an Option Grid PDA for symptomatic uterine fibroids.

### Recruitment

The 5 institutions were selected to participate in our implementation trial because of their inherent diversity, their interest in implementing PDAs, and, in some cases, their experience of practicing shared decision-making. Institutions (from inner city Bronx to rural New Hampshire) treat an ethnically diverse patient population across both urban and rural settings. At each of the 5 institutions, an obstetrics and gynecology specialist was recruited for the role of clinical champion (site principal investigator) for their interest in using PDAs to improve health care delivery. The clinical champions contacted the Epic software analysts and explained the importance of integrating the uterine fibroids Option Grid PDA into the EHR. The clinical champion also introduced software analysts to the UPFRONT study team, which included an experienced Epic technologist employed at EBSCO Health (FA).

### Data Collection

Videoconference meetings were scheduled between the UPFRONT team and the Epic software analysts at each institution. Following the introductory meeting, the Epic software analysts dictated the frequency of the meetings based on their needs for assistance or clarification from the Epic technologist. The software analysts reached out to our study team to schedule meetings. The meetings were a time to collect the work summaries that had been collected by the software analysts, identify any barriers they were facing, and discuss solutions to overcome those barriers. Each meeting was audio-recorded. The transcribed audio recordings and field notes from the meetings provided the data for analysis.

### Consent

The Dartmouth College Committee for the Protection of Human Subjects waived the requirement for the written documentation of informed consent. Standard information regarding the study was provided to the participants. The study team sought verbal consent from the audio recording at the start of videoconference meetings. If verbal consent was not granted by one or more members, then the study team took notes of meeting discussions.

### Analysis

For aim 1, 2 researchers (PS and DS) reviewed meeting transcripts and field notes and documented the steps taken to integrate the Option Grid into Epic. Our description of the steps was reviewed and edited by the Epic technologist (FA) and modifications were made if required. To determine the amount of time required for the integration, we counted the number of months from the original email to the clinical champions to gauge their interest in a possible integration effort to the moment the Option Grid was launched in the site’s Epic environment. For aim 2, we conducted an inductive thematic analysis of the transcripts and field notes. Two researchers (PS and DS) independently coded a sample of transcript pages and then discussed and agreed on a codebook. The finalized codebook (Multimedia Appendix 2) was applied to all the data to highlight the facilitators and barriers to the integration effort. Codes were grouped into different code categories, which were revised and discussed by PS and DS to determine the themes. Coding disagreements were resolved by a third researcher (GE).

## Results

### Overview

A total of 27 meetings were held. These included 25 videoconferences (Zoom, Webex [Cisco], or Skype [Microsoft Corporation]) and 2 telephone meetings. Of the 27 meetings, we were able to record 22 (81%) meetings and collated notes for the remaining 5 (19%). The bidirectional exchange of information between the Epic software analysts and the study team (including the Epic technologist) during the meetings at each institution yielded a total of 183 transcripts or field note pages (total word count: 91,336; see Table 2 for details).

**Table 2:** Details of the 27 meetings that informed the steps to integrate the patient decision aid into the electronic health record and the facilitators and barriers to the integration effort.

Institution and meeting date			Platform		Recorded? (yes or no)		Meeting duration (min:sec)		Meeting personnel
**Dartmouth-Hitchcock Medical Center**									
	January 9, 2019	Zoom		Yes		40:03		3 UPFRONT^a^ study members, 4 EBSCO^b^ Health members, and 2 clinical informatics members and clinicians	
	February 6, 2019	Phone		Yes		44:50		2 UPFRONT study members, 1 clinical champion, 1 Epic technologist, 1 EBSCO Health member, 1 clinical informatics member and clinician, and 2 clinicians	
	July 25, 2019	Webex		Yes		23:39		3 UPFRONT study members and 1 Epic technologist	
	August 9, 2019	Zoom		Yes		5:49		2 UPFRONT study members, 1 Epic technologist, and 1 clinical informatics member and clinician	
**Barnes-Jewish Hospital**									
	February 4, 2019	Zoom		Yes		50:24		3 UPFRONT study members, 1 clinical champion, 1 Epic technologist, 2 EBSCO Health members, and 1 research assistant	
	May 23, 2019	Zoom		Yes		27:25		3 UPFRONT study members, 1 Epic technologist, and 1 Epic software analyst	
	June 6, 2019	Zoom		Yes		35:07		3 UPFRONT study members, 2 Epic software analysts, and 1 Epic technologist	
	February 3, 2020	Webex		Yes		MDNR^c^		1 UPFRONT study member, 1 Epic software analyst, 2 clinicians, and 18 ambulatory operations members	
	February 11, 2020	Webex		Yes		MDNR		1 UPFRONT study member, 1 Epic technologist, 1 Epic software analyst, and 1 interfaces team member	
	March 3, 2020	Webex		Yes		27:35		1 UPFRONT study member, 1 Epic technologist, and 1 Epic software analyst	
**Montefiore Medical Center**									
	January 22, 2019	Zoom		Yes		7:45		2 clinical champions, 1 UPFRONT study member, 1 Epic software analyst, and 3 research assistants	
	January 30, 2019	Zoom		Yes		27:38		2 UPFRONT study members, 2 EBSCO Health members, and 4 Epic software analysts	
	January 14, 2020	Skype		No		—^d^		1 clinical champion, 2 UPFRONT study members, 1 Epic technologist, 2 Epic software analysts, and 2 research assistants	
**Brigham and Women’s Hospital**									
	January 25, 2019	Zoom		Yes		21:59		2 UPFRONT study members and 1 partners operations member	
	February 18, 2019	Zoom		Yes		33:28		2 UPFRONT study members and 2 Epic software analysts	
	March 27, 2019	Zoom		Yes		15:45		2 UPFRONT study members and 2 Epic software analysts	
	May 22, 2019	Zoom		Yes		37:08		3 UPFRONT study members, 1 Epic technologist, 5 Epic software analysts, and 1 research assistant	
	July 29, 2019	Zoom		Yes		18:42		2 UPFRONT study members, 1 Epic technologist, 1 partners operations member, and 2 research assistants	
	January 9, 2020	Webex		Yes		MDNR		1 UPFRONT study member, 1 Epic technologist, and 2 Epic software analysts	
	March 30, 2020	Webex		Yes		MDNR		1 clinical champion, 1 UPFRONT study member, 1 Epic technologist, and 2 Epic software analysts	
	April 24, 2020	Phone		No		—		1 UPFRONT study member, 1 Epic technologist, 1 research assistant, and 2 information security analysts	
	May 28, 2020	Webex		No		—		1 Clinical champion, 1 Epic technologist, 2 Epic software analysts, and 1 research assistant	
	June 5, 2020	Webex		No		—		1 UPFRONT study member, 1 Epic technologist, 2 Epic software analysts, and 1 EBSCO Health member	
	June 23, 2020	Webex		No		—		1 UPFRONT study member, 1 Epic technologist, and 2 Epic software analysts	
**Mayo Clinic**									
	January 23, 2019	Webex		Yes		47:59		1 Clinical champion, 1 UPFRONT study member, and 3 Epic software analysts	
	July 24, 2019	Zoom		Yes		47:33		1 clinical champion, 3 UPFRONT study members, 1 Epic technologist, 3 Epic software analysts, and 1 research assistant	
	January 29, 2020	Webex		Yes		MDNR		1 UPFRONT study member, 1 Epic technologist, and 3 Epic software analysts	

^a^UPFRONT: Uterine Fibroids Options.

^b^EBSCO: Elton B Stephens Company.

^c^MDNR: meeting duration not recorded.

^d^Not available. Participants did not agree to be recorded.

### Aim 1: The Steps Taken to Integrate the PDA Into Epic

We were able to describe the process of integrating the Option Grid PDAs into Epic by identifying 6 common process steps across the institutions (Textboxes 1 and 2).

**Figure 2:**
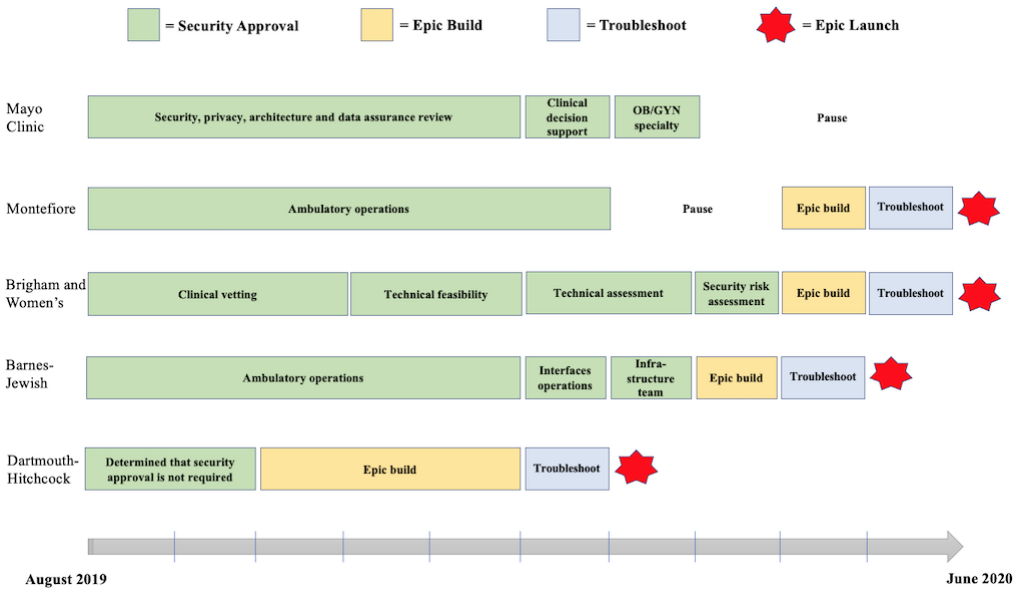
Timeline of the electronic health record integration of the Option Grid patient decision aid. OB/GYN: obstetrics and gynecology.

The timeline for completing the 6 steps varied across institutions, but overall, up to 18 months (January 2019 to June 2020) was required to integrate the Option Grid PDAs into the Epic EHR system. The timeline began in January 2019, which was when all clinical champions received an email to gauge interest in integrating Option Grid into a clinician-facing menu in their Epic system. Although work began to place a button in an agreed location nominated by clinicians at each institution, a policy decision was made by EBSCO Health in August 2019 to use Substitutable Medical Applications and Reusable Technologies (SMART) and Fast Healthcare Interoperability Resources (FHIR) standards and list the application on Epic’s App Orchard to simplify the setup and maintenance of the tool throughout the study period. Owing to the COVID-19 pandemic, integration efforts at 2 institutions were paused to redirect resources to other more pressing initiatives. One institution was able to resume the effort, whereas the other institution had competing priorities and was forced to furlough some personnel involved in the integration process. Thus, we were only able to complete the integration of Option Grid into Epic at 4 of the 5 sites. Figure 2 details the integration timeline following the policy decision, beginning with the security risk assessment (Step 2) and ending with the Option Grid launch (Step 6).

**Steps to integrate the Option Grid patient decision aids into the electronic health record.**

**Step 1: Negotiating task priority with Epic software teams**

- The clinical champions at each site requested the changes into their Epic systems, establishing the clinical benefit of providing easy access to the Option Grid website in a clinician-facing menu location. Epic teams had to reprioritize their tasks, given existing work schedules. In one setting, agreement to reprioritize required negotiation and financial support

**Step 2: Security risk assessment**

- Each institution had different security risk assessment processes, with each requiring departmental approval. Typically, 3 levels of security checks were required, related to the changes in outpatient processes or menus, information flows and dependencies, and communication with third-party tools. Epic has different modules (ie, outpatient, inpatient, and research) and each has its own operations and approval groups. The Ambulatory Operations group is responsible for reviewing requests related to general outpatient workflows (Barnes-Jewish Hospital and Montefiore Medical Center). In the case of Barnes-Jewish Hospital, the integration had to also be approved by the Interfaces Operations group, which manages requests related to any information that gets moved in and out of Epic via interfaces, and the Infrastructure team, which manages the App Orchard. The Mayo Clinic also had 3 levels of security: (1) Security, Privacy, Architecture and Data Assurance reviews new technology that will be integrated into Epic; (2) Clinical Decision Support reviews electronic health record (EHR) change requests that have endorsement from a clinical or practice committee and includes some form of clinical decision support (ie, patient decision aids [PDAs]); and (3) the Obstetrics and Gynecology specialty group, which reviews any new process, procedure, or app that will be integrated into Epic from the clinical perspective. The 4 levels of security at Brigham and Women’s Hospital include (1) the clinical vetting that reviews the study context with 2 clinicians; (2) the technical feasibility of the project evaluated by an Epic team leader, (3) technical assessment (see Step 3 for details); and (4) the security risk assessment that represents an internal process to review the application being integrated into Epic. Security review was considered unnecessary at Dartmouth-Hitchcock because the Option Grid tools were merged with an existing decision support product—HealthDecision. Analysts were particularly focused on data exchange requirements between the Option Grid app and Epic. The lack of protected health information (PHI) transfer was important. Using Substitutable Medical Applications and Reusable Technologies on Fast Healthcare Interoperability Resources to allow approval for the synchronization of the private Option Grid App Orchard app with the institution’s Epic environment (Step 4 for details) standardized the process across all 5 sites

**Step 3: Technical review**

- The Epic software analysts determined the number of personnel and time required for the overall software changes. Once the level of effort, allocation of tasks, and timelines were established, the software analysts were ready for the build

**Step 4: Epic configuration**

- Before commencing the build, all clinical champions indicated their preference for placing the Option Grid button in the patient’s chart under the More menu in their toolbar at the top. Some clinical champions preferred that the button be accessible to the entire institution, whereas others preferred a restricted access to their obstetrics and gynecology department. Our study team’s Epic technologist then developed and shared a build guide with each institution. The guide outlined the study objectives, the Epic-specific configurations required, and described how the Option Grid would not require access to PHI. The Epic software analysts configured access to the Option Grid PDA using Health Level 7 Substitutable Medical Applications and Reusable Technologies on Fast Healthcare Interoperability Resources (see Textbox 2 for further details). The guide contained 4 steps:
  Accessing the app at the Epic App Orchard
  Enabling synchronization of the Option Grid PDA on the App Orchard with the institution’s Epic environment
  Establishing a test environment before launch in a production environment
  Requesting whitelisting of the Option Grid domains so that the app could be accessed within Epic’s EHR menus, tasks, and options. Epic uses the term Hyperspace to describe this view of the software. Launching Option Grid within Hyperspace allows clinicians to have an easy access to the PDAs, without having to navigate to an external website

**Step 5: Troubleshooting**

- The software analysts ensured that menu locations, access requests, and user identification were all functioning as planned. This step represented a final check to ensure that all the Option Grid features were operational

**Step 6: Launch**

- After troubleshooting, the new configuration was migrated to the production environment and the Option Grid was launched. This means that clinicians could access the Option Grid button and be directed to the Option Grid website where they can generate a PDA for their patients

**Health Level 7 Substitutable Medical Applications and Reusable Technologies on Fast Healthcare Interoperability Resources authentication.**

**SMART on FHIR**

- Substitutable Medical Applications and Reusable Technologies (SMART) is an “open, standards-based platform that enables innovators to create applications that seamlessly and securely run across the healthcare system” [35]. Health Level 7 (HL7) is an “industry organization that develops standards for the exchange, integration, sharing and retrieval of EHR information” [36]. HL7 adopted the OAuth2-based SMART App Launch framework as a core interoperability standard. HL7 also developed the Fast Healthcare Interoperability Resources (FHIR) standard to ensure *interoperability, extensibility, and speed* while searching for information across clinical applications [37]. SMART, along with FHIR—collectively referred to as SMART on FHIR—connects third-party apps to Epic, enabling them to reliably and securely launch in Epic’s Hyperspace desktop client [38]. We used SMART on FHIR authentication for the following 3 reasons:
  Improved analytics: allows the tracking of app use so that we can determine the number of eligible patients who received the uterine fibroid Option Grid patient decision aid (PDA)
  Improved control for data access: SMART on FHIR allows a better control of the information shared with third-party apps
  Uses existing authorizations: using FHIR allows clinician access to the Option Grid PDAs in their existing user interface (Epic’s Hyperspace)
- To leverage SMART on FHIR, Epic requires apps to participate in its App Orchard app store. For the UPFRONT (Uterine Fibroids Options) study, EBSCO (Elton B Stephens Company) chose to list Option Grid as a private app on the Epic App Orchard and allowed access by the 5 participating institutions. This also simplified the setup and maintenance of the PDA app during the study period.

### Aim 2: Thematic Analysis of Facilitators and Barriers to EHR Integration

We identified 4 integration facilitators (Textbox 3):

**Facilitators of electronic health record integration.**

**Facilitators**

1. Clinical advocacy: presence of a clinical champion at each institution
2. Electronic health record expertise: presence of an Epic technologist with experience in building apps in Epic
3. Standardization of process: use of Substitutable Medical Applications and Reusable Technologies on Fast Healthcare Interoperability Resources standards
4. Avoidance of protected health information (PHI) data transfer: no exchange of PHI between Option Grid and the institution’s local Epic environment

#### Clinical Advocacy

The clinical champions provided the required professional arguments for integration and served as a gateway to the Epic software analysts and lobbied for prioritization of the task. For instance, the principal investigator at Montefiore Medical Center advocated for the Epic software analysts to prioritize the integration effort:

We are getting it prioritized by the Epic work group with a goal for the summer.

Principal investigator at the Montefiore Medical Center

#### EHR Expertise

Having an EHR expert on our study team with a direct experience of Epic EHR environments and of the app’s requirements at EBSCO was a major facilitator. He developed a *build guide*, which facilitated the integration of Option Grid PDAs in Epic at the 5 settings. Throughout the meetings, the EHR expert supported the software analysts by answering questions, clarifying points of confusion, and providing solutions for any technical issues that arose throughout the process. The following is an example of an exchange between the Epic expert and a software analyst at Barnes-Jewish Hospital during the troubleshooting step of the integration process:

The environment is listed in our App Orchard listing and has the test client ID. Can we test? [launch URL provided] The tokens in the OAuth context values are the same.

Epic technologist

Thank you—I updated the URL item in the record and tested it again. That seemed to work! I am able to open the Uterine Fibroids Option Grid, copy and paste information, open a PDF, and print the PDF.

Barnes-Jewish hospital software analyst

#### Standardization of Process

First, the use of SMART on FHIR standards provided a helpful and officially sanctioned way to better control the information being shared with a third party such as the Option Grid PDA. As described in Textbox 2, the process provided clinicians with single sign-on credentials and enabled us to track the number of times each clinician generated or accessed a uterine fibroid PDA. The Epic technologist also indicated that Epic recommends SMART on FHIR over alternative launch methods such as active guidelines and other URL-based methods:

The reason we went with SMART authentication is because Epic specifically advised us to do so for newer implementations.

Epic technologist

#### Avoidance of Protected Health Information Data Transfer

Placing the Option Grid PDA as a private app in Epic’s App Orchard restricted Option Grid from retrieving protected health information (PHI). Option Grid instructed Epic to block certain features (ie, incoming application programming interface), so their organization would be unable to attain PHI. This arrangement eased the security concerns at each institution. For example, a software analyst at the Mayo Clinic informed us that it would be easier to obtain approval from the security team if PHI was not leaving their Epic environment:

When we bring in new technology it has to go to Security, Privacy, Architecture and Data Assurance (SPAD) review, but if there is no PHI it will get blessed faster.

Mayo software analyst

We identified 3 themes that represented barriers to the integration effort (Textbox 4).

**Barriers of electronic health record integration.**

**Barriers**

1. Commercial third-party autonomy: lack of control over the Option Grid patient decision aid product owned by EBSCO Health
2. Electronic health record updates and maintenance: periodic Epic upgrades causing some delays and functionality issues
3. Unforeseen software problems: found while troubleshooting the app leading to minor delays to launch

#### Commercial Third-Party Autonomy

Although our collaboration with EBSCO was an overall facilitator, it also presented a barrier. EBSCO owns the Option Grid product, and as researchers, we have no influence on product development. Future product development may require additional integration efforts during the life of the study and remain a risk area. For instance, the policy decision in August 2019 to change the integration strategy forced software analysts to adapt and use a different configuration than originally planned. The following is a part of the study team’s communication to each institution, explaining the shift in integration strategy:

EBSCO has been working on enhancing Option Grid and its integration with Epic. This process will utilize SMART and FHIR standards. As a result, we are requesting to delay the integration build in your Epic environment.

UPFRONT study team

#### EHR Updates and Maintenance

Some institutions have regular Epic upgrades and either refrain from integrating apps during those upgrades or impose a freeze on all new software configurations. The software analysts did their best to plan ahead and sidestep this barrier at the final step. The following are 2 quotes that highlight reluctance to launch the button during an upgrade:

Some customers have code freezes that could last more than a month when they have Epic upgrades.

EHR technologist

We generally don’t push out new applications during an Epic upgrade.

Brigham software analyst

#### Unforeseen Software Problems

We experienced unforeseen software-related problems during the troubleshooting steps. In some settings, clinicians were unable to print PDF versions of the tool or copy and paste the script documenting the uterine fibroid options discussed in the patient’s record. These software issues were conveyed to our EHR technologist:

We confirmed that the copy/paste does not work when launching the application.

Dartmouth software analyst

The PDF button did not work. I was still able to navigate elsewhere on the page, but the PDF button was non-responsive.

Brigham software analyst

These issues were quickly resolved through collaboration between the software analysts and the Epic technologist and did not significantly delay the launch of the Option Grid in the site’s Epic environments.

## Discussion

### Principal Findings

The integration of Option Grid PDAs into an EHR such as Epic requires clinical advocacy, a standardized process that avoids using PHI, and expertise to guide the process. Without the support of a clinical champion in each setting, we would not have been able to initiate the process of PDA integration. At the core of the work are issues of security and reassuring the organization that data transfers will not breach security protocols. SMART on FHIR addresses the data security requirements by allowing for a better control of the information being shared with a third party such as Option Grid. The availability of an EHR expert on our study team provided the necessary guidance and reassurance to the existing Epic teams. With all these components and facilitators present, the integration process took up to 18 months to achieve. Barriers were the lack of control over the Option Grid product, EHR updates and maintenance, and unforeseen software problems that caused delays and functionality issues.

### Strengths and Limitations

Our use of a case study method to elicit a real-world, in-depth understanding of the steps required to integrate PDAs into Epic, and the associated barriers and facilitators, is novel and provides new insights. However, this study has limitations. First, we were only able to examine the Epic EHR system, so we do not know if our description of the integration process steps applies to other systems. Second, the Option Grid PDAs do not require the use of PHI data. Some PDAs being developed require the exchange of PHI, which we suspect would prolong integration timelines at many institutions. Third, all the institutions were large academic medical centers with expertise in configuring the Epic EHR system, so we do not know if our findings are applicable to smaller clinical practices that lack such capability. Fourth, we did not conduct a thematic analysis to address our first aim. However, despite the absence of a thematic analysis, we feel like a review of meeting transcripts, with oversight from an Epic technologist, represents the appropriate method to determine the steps to integrate the Option Grid PDA into Epic. Furthermore, it is not known whether EBSCO Health would provide an Epic technologist to other customers or organizations aiming to integrate Option Grid in their Epic. Finally, because of the COVID-19 pandemic, we were only able to integrate the Option Grid PDAs at 4 of the 5 institutions. One institution had to redirect resources to other initiatives and were faced with staffing limitations, which led to a pause in the integration effort. We did not include this as a barrier, considering that under normal circumstances, the institution would be positioned to complete the integration of Option Grid in their Epic.

### Results in Context

To the best of our knowledge, this is the first study to describe SMART on FHIR standards to integrate third-party PDAs, such as Option Grid, into an EHR system. Our results address an important gap outlined by a recent feasibility study that integrated a third-party prostate cancer screening PDA app into the EHR [3]. The authors of that study recognized the potential of SMART on FHIR to standardize secure data exchange and enable integration across a variety of EHRs [3]. However, research has focused on the interoperability of FHIR standards. For instance, a recent review showed how FHIR moved clinical information (medical images and quality metrics) found on different platforms in the EHR into a single platform to streamline the workflow of radiologists [37]. Similarly, another system has used FHIR standards to collect data from multiple sources in the EHR, automate analyses of laboratory test results, and generate easy-to-read reports for patients and their clinicians [39].

For aim 2, a key facilitator of Option Grid PDA integration into Epic systems was the presence of a clinical champion. This aligns with the results of an effort to integrate an EHR-based PDA in the emergency department for concussion and brain injury decisions [40]. They reported the critical need to engage clinicians and other information technology stakeholders [40]. In our case, the clinical champion served as an intermediary between the study team and the Epic software analysts, facilitated prioritization, and identified the EHR menu button location to ensure visibility. Clinicians’ input in the integration process is reported to potentially cause the sustained use of the tools in practice [41,42] and is key to an integration effort, regardless of the format or mode of delivery [14,43-45].

### Implications

Integrating third-party software into EHR systems requires a clinical champion to advocate for the task at the institutional level and an EHR expert who can guide software analyst teams throughout the process. From a policy perspective, implementing SMART on FHIR-compatible servers, which has been done at Duke Medical Center, can improve interoperability and the seamless integration of patient-facing apps [46]. However, the technologies for standardizing the integration of various types of apps, such as Option Grid, do not necessarily mean that they will be used in clinical practice. Integration, though difficult to achieve, seems to be the first step to ensure that clinicians have access to such tools. Providing an integration guide for other organizations to follow and identifying the barriers and facilitators of the process will enable more tools to be integrated into workflows. However, access alone might not lead to use, as observed by others [21]. Future work should include usability, acceptability, or health technology assessments to further evaluate how to trigger clinicians to access and use embedded PDAs [22].

### Conclusions

Integrating the uterine fibroid Option Grid PDA into clinician-facing menus in Epic was an approximately 18-month process, facilitated by a clinical champion who lobbied for the prioritization of the effort at the institutional level, and an EHR expert who guided the Epic software analysts throughout the study. The use of Health Level 7 SMART on FHIR standardized the integration effort, provided clinicians with single sign-on credentials, and more importantly blocked the exchange of PHI between Epic and Option Grid PDAs. Whether integration leads to patient use remains an open question.

## Appendix

Multimedia Appendix 1 The guidelines for the reporting of organizational case studies.

Multimedia Appendix 2 Code hierarchy used to identify the facilitators and barriers of the electronic health record integration effort.

## Abbreviations

AI: artificial intelligence
EBSCO: Elton B Stephens Company
EHR: electronic health record
FHIR: Fast Healthcare Interoperability Resources
LLC: limited liability company
PCORI: Patient-Centered Outcomes Research Institute
PDA: patient decision aid
PHI: protected health information
SMART: Substitutable Medical Applications and Reusable Technologies
UPFRONT: Uterine Fibroids Options

## References

[1] Coylewright M, Keevil JG, Xu K, Dodge SE, Frosch D, Field ME (2020). Pragmatic Study of Clinician Use of a Personalized Patient Decision Aid Integrated into the Electronic Health Record: An 8-Year Experience. Telemed J E Health.

[2] Lenert L, Dunlea R, Del FG, Hall LK (2014). A model to support shared decision making in electronic health records systems. Med Decis Making.

[3] Day FC, Pourhomayoun M, Keeves D, Lees AF, Sarrafzadeh M, Bell D, Pfeffer MA (2019). Feasibility study of an EHR-integrated mobile shared decision making application. Int J Med Inform.

[4] Tejero A, de LTI (2012). Advances and current state of the security and privacy in electronic health records: survey from a social perspective. J Med Syst.

[5] Ratwani RM, Savage E, Will A, Arnold R, Khairat S, Miller K, Fairbanks RJ, Hodgkins M, Hettinger AZ (2018). A usability and safety analysis of electronic health records: a multi-center study. J Am Med Inform Assoc.

[6] Ben-Assuli O (2015). Electronic health records, adoption, quality of care, legal and privacy issues and their implementation in emergency departments. Health Policy.

[7] McGinn T (2016). Putting Meaning into Meaningful Use: A Roadmap to Successful Integration of Evidence at the Point of Care. JMIR Med Inform.

[8] Zhao JY, Song B, Anand E, Schwartz D, Panesar M, Jackson GP, Elkin PL (2017). Barriers, Facilitators, and Solutions to Optimal Patient Portal and Personal Health Record Use: A Systematic Review of the Literature. AMIA Annu Symp Proc.

[9] Elwyn G, Lloyd A, Joseph-Williams N, Cording E, Thomson R, Durand M, Edwards A (2013). Option Grids: shared decision making made easier. Patient Educ Couns.

[10] Tsulukidze M, Grande SW, Gionfriddo MR (2015). Assessing Option Grid® practicability and feasibility for facilitating shared decision making: An exploratory study. Patient Educ Couns.

[11] Seal RP, Kynaston J, Elwyn G, Smith PEM (2014). Using an Option Grid in shared decision making. Pract Neurol.

[12] Stacey D, Légaré F, Lewis K, Barry MJ, Bennett CL, Eden KB, Holmes-Rovner M, Llewellyn-Thomas H, Lyddiatt A, Thomson R, Trevena L (2017). Decision aids for people facing health treatment or screening decisions. Cochrane Database Syst Rev.

[13] Scalia P, Durand M, Berkowitz JL, Ramesh NP, Faber MJ, Kremer JAM, Elwyn G (2019). The impact and utility of encounter patient decision aids: Systematic review, meta-analysis and narrative synthesis. Patient Educ Couns.

[14] Elwyn G, Scholl I, Tietbohl C, Mann M, Edwards AGK, Clay C, Légaré F, van DWT, Lewis CL, Wexler RM, Frosch DL (2013). "Many miles to go …": a systematic review of the implementation of patient decision support interventions into routine clinical practice. BMC Med Inform Decis Mak.

[15] Dobler CC, Sanchez M, Gionfriddo MR, Alvarez-Villalobos NA, Singh Ospina N, Spencer-Bonilla G, Thorsteinsdottir B, Benkhadra R, Erwin PJ, West CP, Brito JP, Murad MH, Montori VM (2019). Impact of decision aids used during clinical encounters on clinician outcomes and consultation length: a systematic review. BMJ Qual Saf.

[16] Furukawa MF, King J, Patel V, Hsiao C, Adler-Milstein J, Jha AK (2014). Despite substantial progress In EHR adoption, health information exchange and patient engagement remain low in office settings. Health Aff (Millwood).

[17] Ankolekar A, Dekker A, Fijten R, Berlanga A (2018). The Benefits and Challenges of Using Patient Decision Aids to Support Shared Decision Making in Health Care. JCO Clin Cancer Inform.

[18] Inselman J, Branda M, Castaneda-Guarderas A, Gionfriddo MR, Zeballos-Palacios CL, Morris MM, Shah ND, Montori VM, LeBlanc A (2016). Uptake and Documentation of the Use of an Encounter Decision Aid in Usual Practice: A Retrospective Analysis of the Use of the Statin/Aspirin Choice Decision Aid. Med Decis Making.

[19] Jouni H, Haddad RA, Marroush TS, Brown S, Kruisselbrink TM, Austin EE, Shameer K, Behnken EM, Chaudhry R, Montori VM, Kullo IJ (2017). Shared decision-making following disclosure of coronary heart disease genetic risk: results from a randomized clinical trial. J Investig Med.

[20] Ye S, Leppin AL, Chan AY, Chang N, Moise N, Poghosyan L, Montori VM, Kronish I (2018). An Informatics Approach to Implement Support for Shared Decision Making for Primary Prevention Statin Therapy. MDM Policy Pract.

[21] Ballard AY, Kessler M, Scheitel M, Montori VM, Chaudhry R (2017). Exploring differences in the use of the statin choice decision aid and diabetes medication choice decision aid in primary care. BMC Med Inform Decis Mak.

[22] Percefull J, Butler J (2020). Improving mammography through effective screening, brief intervention, and referral to treatment at a rural health center. J Am Assoc Nurse Pract.

[23] Denig P, Schuling J, Haaijer-Ruskamp F, Voorham J (2014). Effects of a patient oriented decision aid for prioritising treatment goals in diabetes: pragmatic randomised controlled trial. BMJ.

[24] Schott SL, Xu K, Berkowitz J, Petersen C, Saunders C, Sobti N, Coylewright M (2019). Timing of electronic health record integrated decision aid (IDEA) for stroke prevention in atrial fibrillation matters. Journal of the American College of Cardiology.

[25] Ajeesh S, Luis R (2018). A Comprehensive Electronic Health Record Based Patient Navigation Module Including Technology Driven Colorectal Cancer Outreach and Education. J Cancer Educ.

[26] Wan Q, Makeham M, Zwar NA, Petche S (2012). Qualitative evaluation of a diabetes electronic decision support tool: views of users. BMC Med Inform Decis Mak.

[27] Finkelstein J, Wood J, Crew K, Kukafka R (2017). Introducing a comprehensive informatics framework to promote breast Cancer risk assessment and chemoprevention in the primary care setting. AMIA Summits on Translational Science Proceedings Jul.

[28] Richardson S, Feldstein D, McGinn T, Park LS, Khan S, Hess R, Smith PD, Mishuris RG, McCullagh L, Mann D (2019). Live Usability Testing of Two Complex Clinical Decision Support Tools: Observational Study. JMIR Hum Factors.

[29] Jenssen BP, Shelov ED, Bonafide CP, Bernstein SL, Fiks AG, Bryant-Stephens T (2016). Clinical Decision Support Tool for Parental Tobacco Treatment in Hospitalized Children. Appl Clin Inform.

[30] Kuo AM, Thavalathil B, Elwyn G, Nemeth Z, Dang S (2018). The Promise of Electronic Health Records to Promote Shared Decision Making: A Narrative Review and a Look Ahead. Med Decis Making.

[31] Scalia P, Durand M, Forcino RC, Schubbe D, Barr PJ, O'Brien N, O'Malley AJ, Foster T, Politi MC, Laughlin-Tommaso S, Banks E, Madden T, Anchan RM, Aarts JWM, Velentgas P, Balls-Berry J, Bacon C, Adams-Foster M, Mulligan CC, Venable S, Cochran NE, Elwyn G (2019). Implementation of the uterine fibroids Option Grid patient decision aids across five organizational settings: a randomized stepped-wedge study protocol. Implement Sci.

[32] Yin R (2017). Case Study Research and Applications Design and Methods.

[33] Rodgers M, Thomas S, Harden M, Parker G, Street A, Eastwood A (2016). Developing a methodological framework for organisational case studies: a rapid review and consensus development process. Health Serv Deliv Res.

[34] DynaMed Shared Decisions. EBSCO Health.

[35] What is SMART?. SMART Health IT.

[36] HL7.

[37] Kamel PI, Nagy PG (2018). Patient-Centered Radiology with FHIR: an Introduction to the Use of FHIR to Offer Radiology a Clinically Integrated Platform. J Digit Imaging.

[38] Mandel JC, Kreda DA, Mandl KD, Kohane IS, Ramoni RB (2016). SMART on FHIR: a standards-based, interoperable apps platform for electronic health records. J Am Med Inform Assoc.

[39] Semenov I, Kopanitsa G, Denisov D, Alexandr Y, Osenev R, Andreychuk Y (2018). Patients Decision Aid System Based on FHIR Profiles. J Med Syst.

[40] Melnick ER, Lopez K, Hess EP, Abujarad F, Brandt CA, Shiffman RN, Post LA (2015). Back to the Bedside: Developing a Bedside Aid for Concussion and Brain Injury Decisions in the Emergency Department. EGEMS (Wash DC).

[41] Jetelina KK, Woodson TT, Gunn R, Muller B, Clark KD, DeVoe JE, Balasubramanian BA, Cohen DJ (2018). Evaluation of an Electronic Health Record (EHR) Tool for Integrated Behavioral Health in Primary Care. J Am Board Fam Med.

[42] McCrorie C, Benn J, Johnson OA, Scantlebury A (2019). Staff expectations for the implementation of an electronic health record system: a qualitative study using normalisation process theory. BMC Med Inform Decis Mak.

[43] Scalia P, Elwyn G, Durand M (2017). "Provoking conversations": case studies of organizations where Option Grid™ decision aids have become 'normalized'. BMC Med Inform Decis Mak.

[44] Silvia KA, Sepucha KR (2006). Decision aids in routine practice: lessons from the breast cancer initiative. Health Expect.

[45] Uy V, May SG, Tietbohl C, Frosch DL (2014). Barriers and facilitators to routine distribution of patient decision support interventions: a preliminary study in community-based primary care settings. Health Expect.

[46] Bloomfield RA, Polo-Wood F, Mandel JC, Mandl KD (2017). Opening the Duke electronic health record to apps: Implementing SMART on FHIR. Int J Med Inform.

