# Supplementary material for: Integrating Option Grid Patient Decision Aids in the Epic Electronic Health Record: Case Study at 5 Health Systems
Source: J Med Internet Res. 2021 May 3;23(5):e22766. doi: 10.2196/22766 (PMC8129884; doi:10.2196/22766)
Supplement: Multimedia Appendix 1 [file jmir_v23i5e22766_app1.docx]

Multimedia Appendix 1. The Rodgers *et al* guideline for the reporting of organizational case studies.

| **Reporting Item** |  |
| --- | --- |
| *Describing the design* |  |
| 1. Define the research as a case study | ✓ |
| 2. State the broad aims of the study | ✓ |
| 3. State the research question(s)/hypotheses | ✓ |
| 4. Identify the specific case(s) and justify the selection | ✓ |
| *Describing the data selection* |  |
| 5. Describe how data were collected | ✓ |
| 6. Describe the sources of evidence used | ✓ |
| 7. Describe any ethical considerations and obtainment of relevant approvals, access, and permissions | ✓ |
| *Describing the data analysis* |  |
| 8. Describe the analysis methods | ✓ |
| *Interpreting the results* |  |
| 9. Describe any incoherent shortcomings in the design and analysis and how these might have influenced the findings | ✓ |
| 10. Consider the appropriateness of the methods used for the question and subject matter and why it was that qualitative methods were appropriate. | ✓ |
| 11. Discuss the data analysis | ✓ |
| 12. Ensure that the assertions are sound, neither over- nor under-interpreting the data | ✓ |
| 13. State any caveats about the study | ✓ |
