# Supplementary material for: Integrating Option Grid Patient Decision Aids in the Epic Electronic Health Record: Case Study at 5 Health Systems
Source: J Med Internet Res. 2021 May 3;23(5):e22766. doi: 10.2196/22766 (PMC8129884; doi:10.2196/22766)
Supplement: Multimedia Appendix 2 [file jmir_v23i5e22766_app2.docx]

Multimedia Appendix 2. Code hierarchy used to identify the facilitators and barriers of the EHR integration effort.

|  | **Themes** | **Code Categories** | **Sub-codes** |
| --- | --- | --- | --- |
| Facilitators | Utilization of SMART on FHIR standards | Hyperspace | IE requirements |
|  |  |  | Setup |
|  |  |  | Share links |
|  |  |  | External web browser |
|  |  | Other ways to access OG | Smartshell |
|  |  |  | Xealth |
|  |  |  | Standard Epic functionality |
|  |  |  | Best practice advisories (BPAs) |
|  |  |  | Active guidelines |
|  |  |  | CDS Hooks |
|  |  |  | Infobutton |
|  |  |  | SAML |
|  |  |  | HTTP Get encrypted |
|  |  |  | OBGYN navigator |
|  |  | FHIR/Smart launch | Track usage |
|  |  |  | Recommended by Epic |
|  |  |  | Tokens |
|  |  |  | Integration standardization |
|  |  |  | Smartphrase |
|  |  |  | Single sign-on |
|  |  |  | Click fatigue |
|  |  |  | No data request thru FIHR |
|  |  |  | OAuth 2.0 |
|  | No exchange of PHI between Option Grid and the institution’s local Epic environment. | PHI | Link back to particular patient |
|  |  |  | Exchange between OG and EHR |
|  |  |  | Switch data back into the record |
|  |  |  | Share PHI with OG |
|  |  |  | API |
|  |  |  | Private listing in App Orchard |
|  |  | HIPAA-compliant | De-identified data |
|  |  |  | Retrieval of patient data |
|  |  | Approval from various groups | IRB |
|  |  |  | Medical Records group |
|  | Presence of a clinical champion at each institution. | Prioritization | Present topic to institution |
|  |  |  | Initiate request |
|  |  |  | Resource dedication |
|  |  |  | Approval of next steps |
|  |  |  | Provide study context |
|  |  |  | Funding |
|  |  |  | Completion of security forms |
|  |  | Button launch point | Patient chart |
|  |  |  | Top toolbar |
|  |  |  | Within patient chart |
|  |  |  | Left activity bar |
|  |  |  | HealthPlan tab |
|  |  |  | Pages app tab |
|  |  |  | MORE activities |
|  |  |  | AVS |
|  |  |  | SDM tab |
|  |  |  | Up to date |
|  |  |  | Dotphrase |
|  |  | *Who* gets access? | Entire OBGYN practice |
|  |  |  | Specific clinicians |
|  |  |  | Entire institution |
|  |  |  | Encounter types |
|  | Presence of a Epic technologist with experience in building applications in Epic. | Guide | Integration steps |
|  |  |  | Demonstration of how OG works |
|  |  |  | Liaison to App Orchard TS |
|  |  |  | Build Guide |
|  |  |  | Troubleshooting |
|  |  |  | FDI record reviewer |
| Barriers | Lack of agency to control the Option Grid decision aid product owned by EBSCO. | UF OG | Access to entire suite |
|  |  |  | Access to only UF tool |
|  |  |  | Number of options available |
|  |  | Type of build | SMART on FHIR |
|  |  | HealthDecision | Merger |
|  | Periodic Epic upgrades causing some delays and functionality issues. | Timing | - |
|  |  | EHR version | - |
|  |  | Code freezes | Code changes |
|  | Glitches found when troubleshooting the application leading to minor delays to launch. | PDF button | Printing |
|  |  | Trouble finding UF tool | - |
|  |  | Client ID | - |
|  |  | Error message | - |
|  |  | Workspace FDI build | RiscPacs.Redirector/ ActiveGuidlines |
